# Durability of Response to SARS-CoV-2 BNT162b2 Vaccination in Patients on Active Anticancer Treatment

**DOI:** 10.1001/jamaoncol.2021.4390

**Published:** 2021-08-11

**Authors:** Noa Eliakim-Raz, Amir Massarweh, Amos Stemmer, Salomon M. Stemmer

**Affiliations:** 1 Department of Medicine E, Rabin Medical Center, Beilinson Hospital, Petah Tikva, Israel; 2 Infectious Diseases Unit, Rabin Medical Center, Beilinson Hospital, Petah Tikva, Israel; 3 Sackler Faculty of Medicine, Tel Aviv University, Tel Aviv, Israel; 4 Davidoff Center, Rabin Medical Center, Beilinson Hospital, Petah Tikva, Israel; 5 Department of Oncology, Sheba Medical Center, Tel Hashomer, Ramat Gan, Israel

## Abstract

This cohort study examines the durability of immune response from BNT162b2 vaccination for severe acute respiratory syndrome coronavirus 2 in patients with cancer vs healthy controls.

We recently reported initial findings from a prospective cohort study which evaluated the antispike (anti-S) IgG antibody response to the SARS-CoV-2 BNT162b2 messenger RNA (mRNA) vaccine (BioNTech-Pfizer) in patients with solid tumors on active anticancer treatment vs healthy controls.^1^ After a median of approximately 5.5 weeks from the second vaccine dose, 90% of the patients with cancer (90/102) and 100% of the healthy controls (78/78) were seropositive, and the median IgG titer in the patients was significantly lower than that in the controls:1931 (interquartile range [IQR], 509-4386) AU/mL vs 7160 (IQR, 3129-11241) AU/mL; *P* < .001.^1^

Herein, we describe the anti-S response in the patients with cancer vs the controls approximately 4 months after the second vaccine dose.

## Methods

Study design, eligibility criteria, and anti-S IgG evaluation have been previously reported.^1^ The study was approved by the ethics committee of Rabin Medical Center. All participants provided written informed consent.

Statistical analyses were performed as previously described.^1^ A *P* value <.05 was considered significant. Statistical analysis was performed using R (version 4.0.2, R Foundation).^2^

## Results

The previous analysis included 102 patients with cancer and 78 healthy control patients.^1^ The current analysis included 95 of 102 patients (5 died, 2 withdrew) and 66 of 78 controls (12 withdrew). Baseline characteristics of the 95 patients and 66 controls are presented in the Table.

**Table.** Cohort Demographic and Baseline Characteristics

Characteristic	No. (%)	
	Patients with cancer	Controls
No.	95	66
Age, median (IQR), y	65 (56-72)	62 (50-70)
Sex		
Men	55 (58)	21 (32)
Women	40 (42)	45 (68)
Cancer type		
Gastrointestinal	25 (26)	NA
Lung	24 (25)	
Breast	17 (18)	
Other^a^	12 (13)	
Brain	9 (9)	
Genitourinary	8 (8)	
Treatment		
Chemotherapy	27 (28)	NA
Immunotherapy	20 (21)	
Chemotherapy + biological therapy	19 (20)	
Chemotherapy + immunotherapy	13 (14)	
Biological therapy	11 (12)	
Immunotherapy + biological therapy	5 (5)	
Days postvaccination, median (IQR)		
Previous analysis^b^	38 (32-43)	40 (33-45)
Current analysis	123 (116-129)	124 (119-134)
IgG titer, median (IQR), AU/mL		
Previous analysis^b^	1957 (488-4384)^c^	7160 (3082-11 036)^d^
Current analysis^e^	417 (136-895)^c^	1220 (588-1987)^d^

Abbreviations: IQR, interquartile range; NA, not applicable.

^a^ Other cancer types included cervix uteri squamous cell carcinoma, desmoid-type fibromatosis, melanoma, mucoepidermoid carcinoma, nasopharynx squamous cell carcinoma, nonmelanoma skin squamous cell carcinoma, osteosarcoma, thymoma, and thyroid anaplastic carcinoma.

^b^ The analysis included the data from the previously published report,^1^ using the 95 patients and 66 controls who comprise the current cohort.

^c^ *P* < .001 for comparing IgG values between the previous and current analyses within the patient group (Wilcoxon signed-rank test).

^d^ *P* < .001 for comparing IgG values between the previous and current analyses within the control group (Wilcoxon signed-rank test).

^e^ *P* < .001 for comparing IgG values between the patients and the controls (Wilcoxon rank sum test).

After a median (IQR) of 123 (116-129) days from the second vaccination, 83 patients (87%) and all the controls (100%) were seropositive for anti-S IgG antibodies. The median titer levels in the patients with cancer was significantly lower than those in the control group (417 [IQR, 136-895] AU/mL vs 1220 [IQR, 588-1987] AU/mL; *P* < .001) (Table; Figure, A). Evaluating the IgG titers by tumor type and anticancer treatment demonstrated a 3.6-fold range in median titer values across tumor types and a wider range (8.8-fold) across treatment types. The lowest titers were observed with immunotherapy plus chemotherapy/biological therapy (median [IQR], 94.4 [49.4-191] AU/mL/147 [62.8-339] AU/mL). In an exploratory multivariable analysis, the only variable significantly associated with lower IgG titers was treatment with chemotherapy plus immunotherapy and immunotherapy plus biological therapy.

**Figure.**
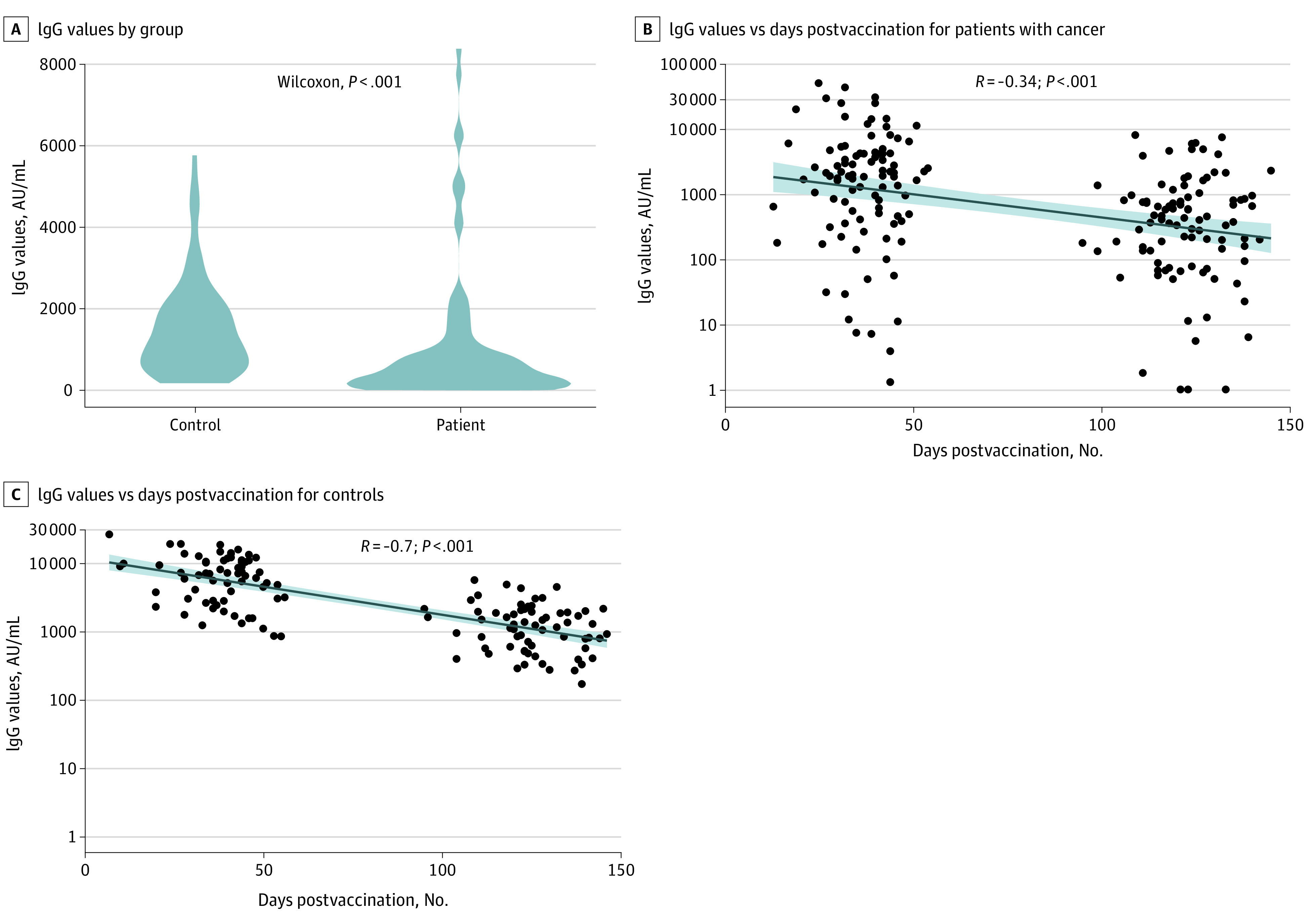
Immunoglobulin G (IgG) Values of Study A, IgG values by group. B, Scatter plot of IgG values vs days postvaccination for patients with cancer. C, Scatter plot of IgG values vs days postvaccination for controls. In panels B and C, the shaded areas indicate 95% CIs and the black dots represent individual participants.

Of the 12 seronegative patients, 8 were seronegative in the previous analysis. One patient with breast cancer who was seronegative in the previous analysis, was no longer on active therapy in the current analysis and became seropositive.

Evaluating the IgG titer as a function of the time between the second vaccine dose and the blood sample drawn from each patient demonstrated a significant negative linear correlation for the patients (*R* = −0.34, *P* < .001) and the controls (*R* = −0.70, *P* < .001) (Figure, B and C).

## Discussion

The seropositivity rate among the patients with cancer remained high (87%) approximately 4 months after the second BNT162b2 vaccination dose. The median IgG titer in the patients and the controls decreased over time. Notably, in both the previous^1^ and the current analysis, the IgG titers were statistically significantly lower in the patients with cancer vs the controls.

Data on the durability of protection after vaccination are limited for healthy participants and lacking for oncological patients. Elevated antibody levels persisting 3 months after the second dose of mRNA-1273 vaccine (Moderna) were reported in 34 participants, although a slight decrease in antibody levels was reported.^3^ Interim results from a phase 3 trial of the mRNA-1273 vaccine in 33 healthy adults demonstrated that the antibody activity remained high in all age groups after approximately 7 months.^4^ Although the correlation between antibody levels after vaccination and clinical protection is yet to be proven, the accumulating evidence supports antibody response as a potential correlate of disease protection.^5^ Long-term cellular memory could call into question the need for a third BNT162b2 booster dose. Study limitations include lack of cellular immunity testing and/or neutralizing antibody testing.
